# Blockade of NF-κB Translocation and of RANKL/RANK Interaction Decreases the Frequency of Th2 and Th17 Cells Capable of IL-4 and IL-17 Production, Respectively, in a Mouse Model of Allergic Asthma

**DOI:** 10.3390/molecules26113117

**Published:** 2021-05-23

**Authors:** Izabela Gregorczyk, Agnieszka Jasiecka-Mikołajczyk, Tomasz Maślanka

**Affiliations:** Department of Pharmacology and Toxicology, Faculty of Veterinary Medicine, University of Warmia and Mazury in Olsztyn, Oczapowskiego Street 13, 10-719 Olsztyn, Poland; igrekk.y@gmail.com (I.G.); agnieszka.jasiecka@uwm.edu.pl (A.J.-M.)

**Keywords:** allergic asthma, NF-κB and RANKL/RANK/OPG pathways, cytokines

## Abstract

The main purpose of this study was to investigate whether the blockade of the interaction between the receptor activator of nuclear factor-κB (NF-ĸB) ligand (RANKL) and its receptor RANK as well as the blockade of NF-κB inhibitor kinase (IKK) and of NF-κB translocation have the potential to suppress the pathogenesis of allergic asthma by inhibition and/or enhancement of the production by CD4^+^ and CD8^+^ T cells of important cytokines promoting (i.e., IL-4 and IL-17) and/or inhibiting (i.e., IL-10 and TGF-β), respectively, the development of allergic asthma. Studies using ovalbumin(OVA)-immunized mice have demonstrated that all the tested therapeutic strategies prevented the OVA-induced increase in the absolute number of IL-4- and IL-17-producing CD4^+^ T cells (i.e., Th2 and Th17 cells, respectively) indirectly, i.e., through the inhibition of the clonal expansion of these cells in the mediastinal lymph nodes. Additionally, the blockade of NF-κB translocation and RANKL/RANK interaction, but not IKK, prevented the OVA-induced increase in the percentage of IL-4-, IL-10- and IL-17-producing CD4^+^ T cells. These latter results strongly suggest that both therapeutic strategies can directly decrease IL-4 and IL-17 production by Th2 and Th17 cells, respectively. This action may constitute an important mechanism underlying the anti-asthmatic effect induced by the blockade of NF-κB translocation and of RANKL/RANK interaction. Thus, in this context, both these therapeutic strategies seem to have an advantage over the blockade of IKK. None of the tested therapeutic strategies increased both the absolute number and frequency of IL-10- and TGF-β-producing Treg cells, and hence they lacked the potential to inhibit the development of the disease via this mechanism.

## 1. Introduction

Although glucocorticosteroids are still the mainstay of asthma therapy, they are not always sufficiently efficacious and their use can be associated with side effects. Moreover, glucocorticoid insensitivity presents a profound management problem in patients with asthma because conventional therapies are not effective [1]. The treatment of patients with steroid-resistant asthma will require novel therapies tailored to this specific subgroup of patients. Therefore, researchers are looking for new targets for allergic asthma therapy. Nuclear factor-κB (NF-κB) is considered to be a promising target for the development of a novel therapeutic strategy in asthma treatment because allergic airway inflammation (AAI) is mediated by the NF-κB pathway [2]. Therefore, the down-regulation of this pathway may be an effective and rational approach for the treatment of allergic asthma.

Typically, the receptor activator of NF-ĸB (RANK) ligand (RANKL)/RANK/osteoprotegerin (OPG) system is associated with bone homeostasis. However, RANKL/RANK signaling is important, among others, in lymph-node development, T cell activation and tolerance induction [3]. Moreover, this pathway is also implicated in the induction of the production of proinflammatory cytokines and chemokines [4,5]. The results of our recent studies imply that the blockade of the interaction between RANKL and RANK can be regarded as a novel therapeutic strategy in the treatment of allergic asthma [6]. In this study, we compared efficacies of the blockade of RANKL/RANK interaction as well as the blockade of NF-κB inhibitor kinase (IKK) and of NF-κB translocation to the nucleus with respect to the reduction/prevention of the development of a mouse model of AAI. The blockade of each of these targets fully prevented the development of AAI. The anti-proliferative action on allergen-induced CD4^+^ effector T (Teff) cell proliferation in the mediastinal lymph nodes (MLNs) seems to constitute the leading mechanism responsible for this effect. Thus, all the tested therapeutic strategies adhere to the principal rule of medicine that the development of a disease must be stopped at the earliest possible event during its pathogenesis. Apart from this, however, an effective anti-asthmatic drug should have the potential to halt/inhibit the development of allergic asthma at the next stages of its pathogenesis. If the development of the disease gets out of control at an earlier stage of pathogenesis, it should be broken at subsequent stages. Thus, these additional mechanisms should serve as ‘a safety fuse’ controlling the disease development process.

NF-κB induces the expression of various pro-inflammatory cytokines, including IL-4 [7] and IL-17 [8], which are the cytokines pivotal to the development of immune and inflammatory responses in asthma. This study was performed to resolve whether the blockade of IKK, NF-κB translocation and of RANKL/RANK interaction could lead to the abolition of the production of IL-4 and IL-17 by CD4^+^ and CD8^+^ Teff cells and/or induction of the production of IL-10 and TGF-β by Foxp3^+^CD25^+^CD4^+^ regulatory T (Treg) cells. For this purpose, the CD4^+^ T cell population was subdivided into Treg (i.e., Foxp3^+^CD25^+^CD4^+^) and non-Treg (the remaining CD4^+^ T cells) cells. The non-Treg CD4^+^ T cell subset was equated with Teff cells because this population predominantly comprises such type of cells. IL-4- and IL-17-producing Teff cells should be identified as T helper type 2 (Th2) and T helper type 17 (Th17) cells. Th2 and Th17 cells and their hallmark cytokines, i.e., IL-4 and IL-17, respectively, play crucial roles in the pathogenesis of allergic asthma. IL-4 is produced mainly by activated Th2 cells and is essential for driving the differentiation of naïve T cells into Th2 cells [9]. Following antigen presentation to naïve T cells in the MLNs, specific CD4^+^ T cells are activated, expanded and differentiated into Th2 cells, and afterwards they migrate to the lungs and orchestrate pulmonary immune responses [10]. Th17 cells also play a key role in promoting and maintaining airway inflammation. IL-17 is considered to be the main proinflammatory cytokine involved in the pathogenesis of allergic asthma; it plays a critical role in neutrophil and eosinophil recruitment to the lungs [11]. Although the central role in the pathogenesis of asthma is played by CD4^+^ T cells, numerous studies prove that CD8^+^ T cells also participate in the development of the disease [12,13,14]. IL-4- and IL-17-producing CD8+ T cells, i.e., T cytotoxic type 2 (Tc2) and T cytotoxic type 17 (Tc17) cells, respectively, are particularly involved in the pathogenesis of asthma [15,16]. Taking all of the above into consideration, the current study verified the hypothesis that the blockade of IKK, NF-κB translocation and of RANKL/RANK interaction can lead to the abolition of IL-4 and IL-17 production by CD4^+^ and CD8^+^ Teff cells. 

Foxp3-expressing Treg cells function as a natural ‘braking system’, counteracting the development of allergic asthma [17]. To a large extent, this effect is mediated by the production of IL-10 and TGF-β [18], which are the key anti-inflammatory and immunosuppressive cytokines. IL-10 especially plays an important inhibitory role—via the inhibition of many effector cells and disease processes—in the pathogenesis of asthma [19]. In light of that, the up-regulation of IL-10 and TGF-β production by Treg cells should translate into a reduction in the development of immune and inflammatory responses in asthma. Therefore, we deemed it worthwhile to verify the hypothesis that the blockade of IKK, NF-κB translocation and of RANKL/RANK interaction might induce the production of IL-10 and TGF-β by Treg cells. Noteworthy, the available literature lacks data concerning this issue.

To recapitulate, the essential purpose of this study was to determine whether all the tested therapeutic strategies, regardless of their inhibitory influence on the clonal expansion of CD4^+^ Teff cells in the MLNs, have the capability to suppress the pathogenesis of allergic asthma at its later stage by inhibition and/or enhancement of the production by CD4^+^ and CD8^+^ T cells of important cytokines promoting (i.e., IL-4 and IL-17) and/or inhibiting (i.e., IL-10 and TGF-β), respectively, the development of allergic asthma. Another aim of this study was to compare in this regard the efficacies of the blockade of IKK, NF-κB translocation and of RANKL/RANK interaction both mutually and with glucocorticosteroid treatment. Such comparisons can shed light on the therapeutic value of the tested therapeutic strategies and resolve the question of whether they would have any advantage over the standard treatment in the scope covered by this study.

## 2. Results

### 2.1. DHMEQ and OPG Prevent OVA-Induced Increase in the Relative and Absolute Counts of IL-4-, IL-10- and IL-17-Producing Non-Treg CD4^+^ T Cells in the Lungs and Administration of BMS Prevents the Absolute, but Not Relative, Increase in the Number of These Cells

Ovalbumin(OVA)-induced effect: OVA immunization led to a significant change in the value of a parameter in vehicle-treated mice (OVA group) compared with vehicle-treated, non-immunized mice (phosphate buffered saline (PBS) group). 

Prevention of OVA-induced effect: the value of a parameter was significantly lower in methylprednisolone(MP)- and/or BMS-345541(BMS)- and/or dehydroxymethylepoxyquinomicin(DHMEQ)- and/or osteoprotegerin(OPG)-treated OVA-immunized animals than in the OVA group, and did not differ significantly from the value of this parameter achieved in the PBS group.

**Figure 1:**
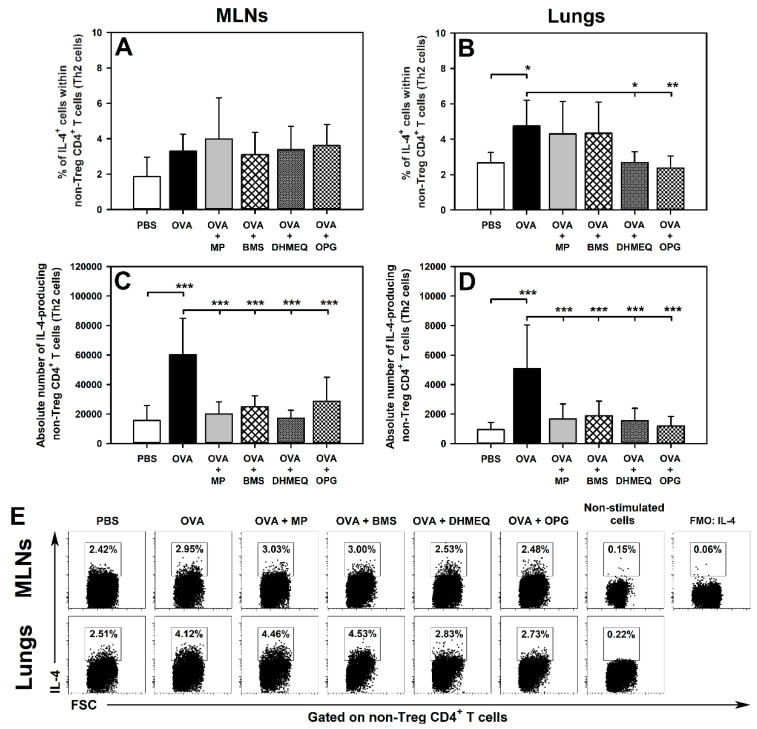
Effect of inhibitors of IKK (BMS-345541 (BMS)), NF-κB translocation (dehydroxymethylepoxyquinomicin (DHMEQ)) and RANK/RANK-L interaction (osteoprotegerin (OPG)), and glucocorticosteroid (methylprednisolone (MP)) administration on the number of IL-4-producing non-Treg CD4^+^ T cells. The relative count (**A**,**B**) is expressed as a percentage of IL-4-producing cells within non-Foxp3^+^CD25^+^ CD4^+^ T cells. The absolute count (**C**,**D**) represents the number of IL-4-producing non-Foxp3^+^CD25^+^ CD4^+^ T cells per MLN or lung sample collected from individual mice. * *p* < 0.05, ** *p* < 0.01, *** *p* < 0.001. Examples of dot plot cytograms showing the distribution of IL-4-producing and non-producing cells within non-Treg CD4^+^ T cells (**E**).

**Figure 2:**
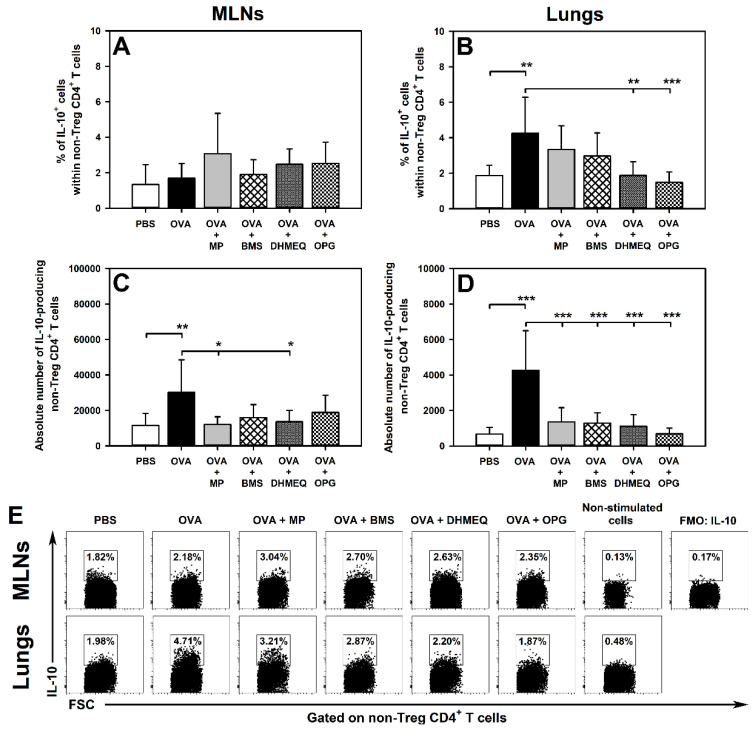
Effect of inhibitors of IKK (BMS-345541 (BMS)), NF-κB translocation (dehydroxymethylepoxyquinomicin (DHMEQ)) and RANK/RANK-L interaction (osteoprotegerin (OPG)), and glucocorticosteroid (methylprednisolone (MP)) administration on the number of IL-10-producing non-Treg CD4^+^ T cells. The relative count (**A**,**B**) is expressed as a percentage of IL-10-producing cells within non-Foxp3^+^CD25^+^ CD4^+^ T cells. The absolute count (**C**,**D**) represents the number of IL-10-producing non-Foxp3^+^CD25^+^ CD4^+^ T cells per MLN or lung sample collected from individual mice. * *p* < 0.05, ** *p* < 0.01, *** *p* < 0.001. Examples of dot plot cytograms showing the distribution of IL-10-producing and non-producing cells within non-Treg CD4^+^ T cells (**E**).

**Figure 3:**
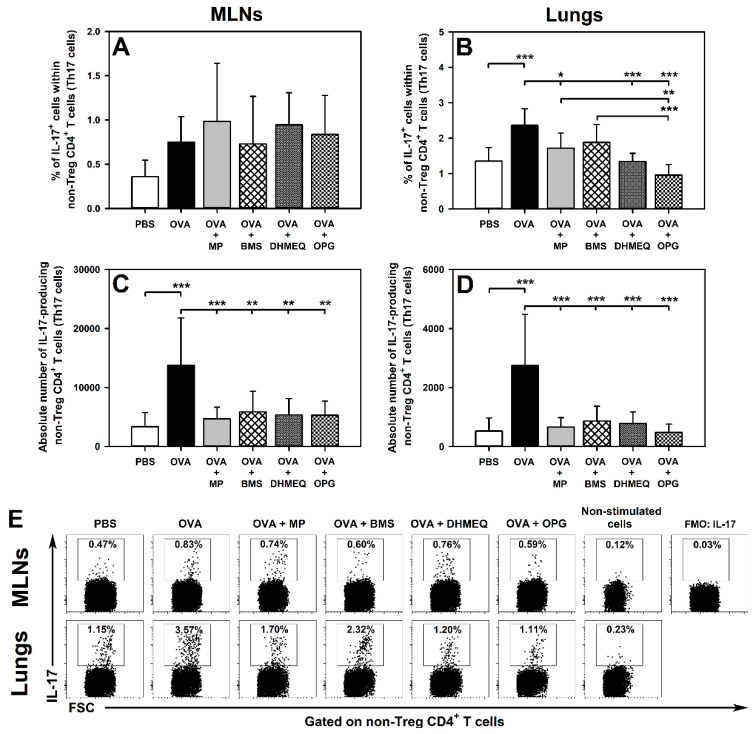
Effect of inhibitors of IKK (BMS-345541 (BMS)), NF-κB translocation (dehydroxymethylepoxyquinomicin (DHMEQ)) and RANK/RANK-L interaction (osteoprotegerin (OPG)), and glucocorticosteroid (methylprednisolone (MP)) administration on the number of IL-17-producing non-Treg CD4^+^ T cells. The relative count (**A**,**B**) is expressed as a percentage of IL-17-producing cells within non-Foxp3^+^CD25^+^ CD4^+^ T cells. The absolute count (**C**,**D**) represents the number of IL-17-producing non-Foxp3^+^CD25^+^ CD4^+^ T cells per MLN or lung sample collected from individual mice. * *p* < 0.05, ** *p* < 0.01, *** *p* < 0.001. Examples of dot plot cytograms showing the distribution of IL-17-producing and non-producing cells within non-Treg CD4^+^ T cells (**E**).

Immunization with OVA induced an increase in the percentage of IL-4-, IL-10- and IL-17-producing cells within non-Treg CD4^+^ T cells in the lungs (Figure 1B,E, Figure 2B,E, and Figure 3B,E, respectively). All these effects were prevented by the treatment with DHMEQ and OPG (Figure 1B,E, Figure 2B,E, and Figure 3B,E), while MP prevented only the latter one (Figure 3B,E). The value of these parameters in the lungs of mice treated with BMS did not differ significantly from those achieved in both PBS and OVA groups (Figure 1B,E, Figure 2B,E, and Figure 3B,E). Neither immunization with OVA nor co-administration of all the tested agents affected the percentage of IL-4-, IL-10- and IL-17-producing cells within non-Treg CD4^+^ T cells in the MLNs (Figure 1A,E, Figure 2A,E, and Figure 3A,E, respectively). 

The research showed that OVA immunization led to a significant increase in the absolute number of IL-4-, IL-10- and IL-17-producing non-Treg CD4^+^ T cells in the MLNs (Figure 1C, Figure 2C and Figure 3C, respectively) and lungs (Figure 1D, Figure 2D and Figure 3D, respectively). Almost all these effects were fully prevented by the treatment with MP, BMS, DHMEQ and OPG (Figure 1C,D, Figure 2C,D, and Figure 3C,D). The only exception was the absolute number of IL-10-producing non-Treg CD4^+^ T cells in the MLNs of BMS- and OPG-treated mice. However, the absolute number of these cells in both groups did not differ significantly from the value of this parameter achieved in both PBS and OVA groups (Figure 2C). Such results may suggest that the OVA-induced increase in the absolute number of IL-10-producing non-Treg CD4^+^ T cells in the MLNs was limited to some extent by treatment with BMS and OPG. Moreover, this is also indicated by the fact that the treatment with these agents fully prevented OVA-induced infiltration of the lungs by IL-10-producing non-Treg CD4^+^ T cells.

### 2.2. BMS, DHMEQ and OPG Prevent OVA-Induced Increase in the Absolute Counts of IL-10- and TGF-β-Producing Treg Cells in the Lungs

**Figure 4:**
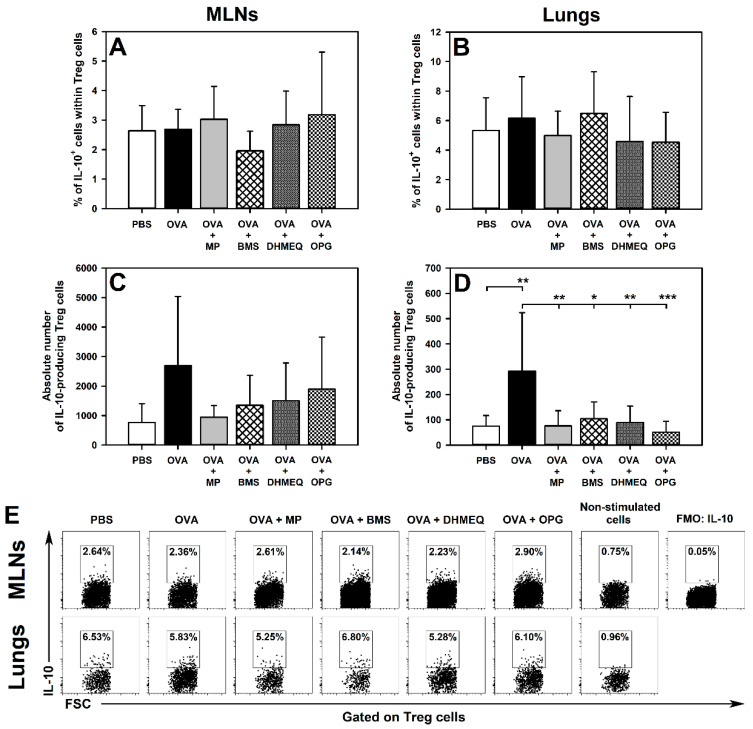
Effect of inhibitors of IKK (BMS-345541 (BMS)), NF-κB translocation (dehydroxymethylepoxyquinomicin (DHMEQ)) and RANK/RANK-L interaction (osteoprotegerin (OPG)), and glucocorticosteroid (methylprednisolone (MP)) administration on the number of IL-10-producing Treg cells. The relative count (**A**,**B**) is expressed as a percentage of IL-10-producing cells within Foxp3^+^CD25^+^CD4^+^ T cells. The absolute count (**C**,**D**) represents the number of IL-10-producing Foxp3^+^CD25^+^CD4^+^ T cells per MLN or lung sample collected from individual mice. * *p* < 0.05, ** *p* < 0.01, *** *p* < 0.001. Examples of dot plot cytograms showing the distribution of IL-10-producing and non-producing cells within Treg cells (**E**).

**Figure 5:**
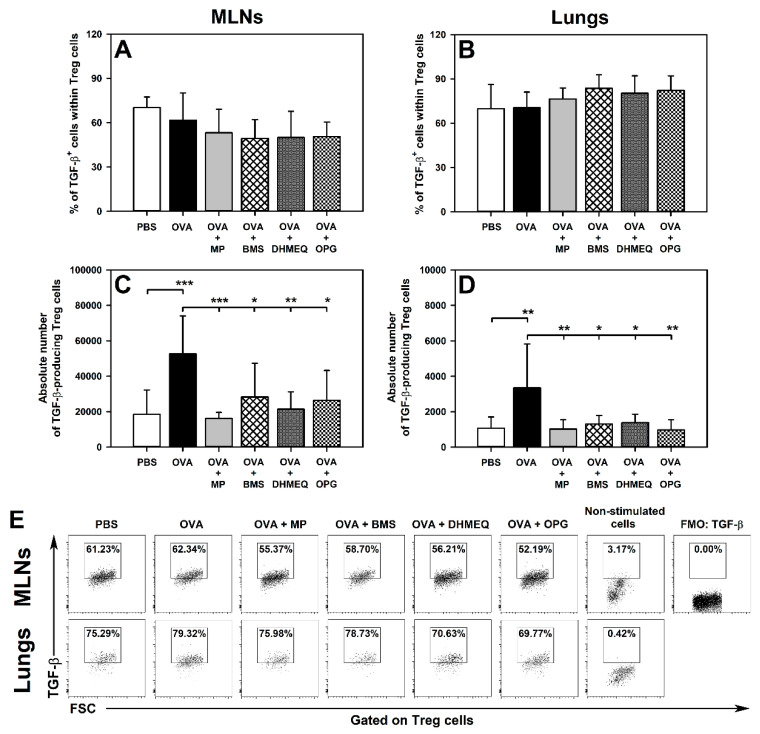
Effect of inhibitors of IKK (BMS-345541 (BMS)), NF-κB translocation (dehydroxymethylepoxyquinomicin (DHMEQ)) and RANK/RANK-L interaction (osteoprotegerin (OPG)), and glucocorticosteroid (methylprednisolone (MP)) administration on the number of TGF-β-producing Treg cells. The relative count (**A**,**B**) is expressed as a percentage of TGF-β-producing cells within Foxp3^+^CD25^+^CD4^+^ T cells. The absolute count (**C**,**D**) represents the number of TGF-β-producing Foxp3^+^CD25^+^CD4^+^ T cells per MLN or lung sample collected from individual mice. * *p* < 0.05, ** *p* < 0.01, *** *p* < 0.001. Examples of dot plot cytograms showing the distribution of TGF-β-producing and non-producing cells within Treg cells (**E**).

Immunization with OVA and co-administration of MP, BMS, DHMEQ and OPG did not affect the percentage of IL-10- (Figure 4A,B,E) and TGF-β-producing cells (Figure 5A,B,E) within the Treg subset in the MLNs and lungs, or the absolute number of IL-10-producing Treg cells in the MLNs (Figure 4C). However, immunization induced a significant increase in the absolute number of IL-10- (Figure 4D) and TGF-β-producing Treg cells (Figure 5D) in the lungs. Additionally, the absolute number of TGF-β-producing Treg cells was found to be elevated in the MLNs of untreated OVA-immunized mice (Figure 5C). The results of our research demonstrated that all these effects were fully prevented by the treatment with MP, BMS, DHMEQ and OPG (Figure 4D and Figure 5C,D).

### 2.3. BMS, DHMEQ and OPG Prevent OVA-Induced Increase in the Absolute Counts of IL-4- and IL-17-Producing CD8^+^ T Cells in the Lungs

**Figure 6:**
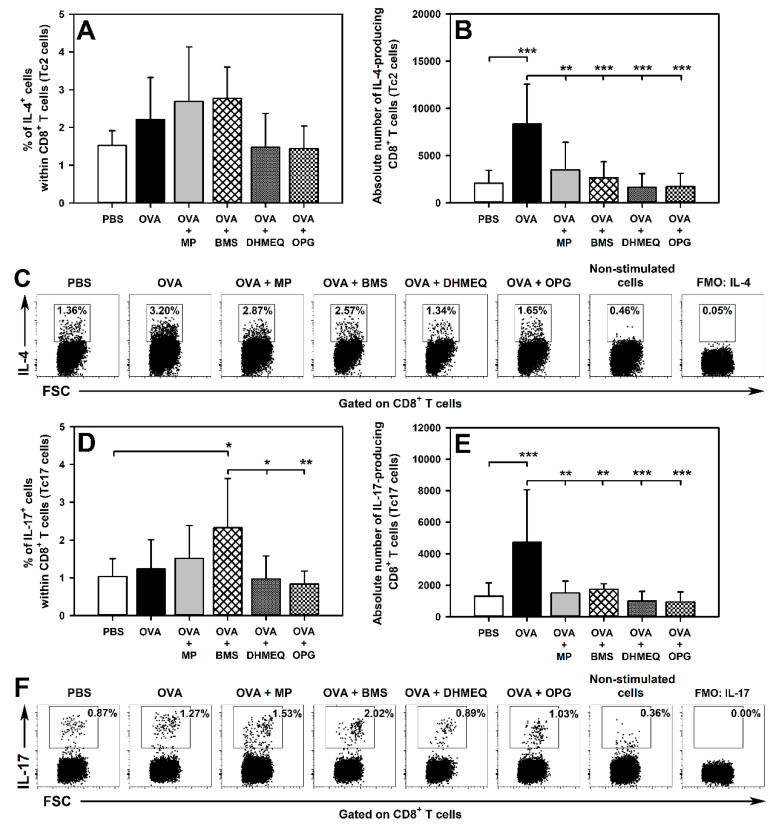
Effect of inhibitors of IKK (BMS-345541 (BMS)), NF-κB translocation (dehydroxymethylepoxyquinomicin (DHMEQ)) and RANK/RANK-L interaction (osteoprotegerin (OPG)), and glucocorticosteroid (methylprednisolone (MP)) administration on the number of IL-4- and IL-17-producing CD8^+^ T cells. The relative count (**A**,**D**) is expressed as a percentage of IL-4- or IL-17-producing cells within CD8^+^ T cells. The absolute count (**B**,**E**) represents the number of IL-4- or IL-17-producing CD8^+^ T cells per lung sample collected from individual mice. * *p* < 0.05, ** *p* < 0.01, *** *p* < 0.001. Examples of dot plot cytograms showing the distribution of IL-4- or IL-17-producing and non-producing cells within CD8^+^ T cells (**C** and **F**, respectively).

This study did not find any effect of immunization with OVA and co-administration of MP, DHMEQ and OPG on the percentage of IL-4- (Figure 6A,C) and IL-17-producing cells (Figure 6D,F) within the CD8^+^ T cell subset in the lungs. However, we found that OVA immunization led to a significant increase in the absolute number of IL-4- (Figure 6B) and IL-17-producing CD8^+^ T cells (Figure 6E) in the lungs and the treatment with all the tested agents prevented this effect (Figure 6B,E). Interestingly, the percentage of IL-17-producing CD8^+^ T cells was significantly higher in the BMS group compared with the non-immunized mice, and with DHMEQ- and OPG-treated OVA-immunized mice (Figure 6D,F). However, the scale of this effect was relatively small, and it was not reflected by the absolute cell number, thus it is doubtful whether it has any clinical importance, despite the determined statistical significance. IL-4- and IL-17-producing CD8^+^ T cells were not detectable in the MLNs.

## 3. Discussion

This paper is the continuation of our earlier comparative studies concerning the blockade of IKK, NF-κB translocation and of RANKL/RANK interaction as novel therapeutic strategies in the treatment of allergic asthma [6]. The essence of these investigations was to identify whether all these therapeutic strategies, regardless of the inhibitory effect on the clonal expansion of CD4^+^ Teff cells in the MLNs, have the potential to break the pathogenesis of allergic asthma at its later stage by abolition and/or induction of the production by T cells of important cytokines promoting and/or inhibiting, respectively, the development of the disease. 

The measurement of cytokine levels is a typical parameter used to evaluate the cytokine production. Moreover, quantitative assessment of the percentage of cytokine-producing cells also represents a valuable tool to investigate this issue, and was used in our [20,21] and other studies [22,23]. This assessment enables the identification of the effects of tested agents on cytokine production with respect to particular cell subsets including, among others, Treg cells. However, it should be noted that a course of allergic asthma involves the proliferation, apoptosis and redistribution (i.e., lung infiltration) of CD4^+^ T cells in different immune compartments, and these processes cannot be captured and measured with the help of percentage analysis of T cell subsets. Therefore, in this case, in order to provide a relatively complete picture of studied agents on the number of cytokine-producing cells, it was necessary to determine the absolute count of these cells in the MLNs and lungs of OVA-immunized mice, which is in inductive and effector compartments, respectively, associated with allergic asthma. Moreover, a comparative analysis of the percentage and absolute data enables, to some extent, the distinction between direct and indirect effect on the number of cytokine-producing T cells. The terms ‘direct’ and ‘indirect’ effect may have various meanings depending on the context in which they are used. These terms have the same meaning in this paper as in previous work [21], i.e., they are defined as follows: the direct effect is associated with the impact on the cell’s ability to produce a cytokine, whereas the indirect effect arises from the influence on the expansion or depletion of potential cytokine producers. Thus, the direct effect arises when a cell acquires or loses the ability to produce a given cytokine under the influence of a specific substance. A proper way to identify possible direct effects is the determination of the percentage of cytokine-producing cells within the relevant cell subset under in vitro stimulation. As the wells are seeded with the same number of cells producing a given cytokine, a change in the percentage of cells producing this cytokine indicates that the tested substance either abolishes or induces the ability to produce this cytokine in the tested cells. The indirect effect appears when a given substance does not affect the cell’s ability to produce the cytokine in question but influences the count of potential producers of this cytokine (as a result of its impact on the proliferation, redistribution or apoptosis of cytokine producers). In the nature of things, a change in the number of cytokine producers will translate into a change in the number of cytokine-producing cells and will eventually affect the level of the produced cytokine. Most studies do not take the above-discussed distinction into consideration. Hence, a secondary aim of this study was to draw attention to this important aspect of research outcome interpretation.

In the present study, immunization led to an increase in the absolute number of IL-4- and IL-17-producing CD4^+^ Teff cells in the MLNs but did not affect the percentages of these cells. Thus, by triggering the clonal expansion of CD4^+^ Teff cells in the MLNs, immunization induced the expansion of IL-4 and IL-17 producers but did not induce the production of these cytokines per se. In turn, not only the absolute number but also the percentage of IL-4 and IL-17-producing CD4^+^ Teff cells were significantly higher in the lungs of untreated immunized mice than in the lungs of non-immunized mice. Thus, immunization caused the infiltration of the lungs by the cytokine producers expanded in the MLNs and induced the ability to produce IL-4 and IL-17 in these cells. The blockade of IKK, NF-κB translocation or of RANKL/RANK interaction as well as the glucocorticosteroid treatment prevented the OVA-induced increase in the absolute counts of IL-4- and IL-17-producing CD4^+^ Teff in the MLNs and lungs. These results fully corroborate our previous findings [6] that the prevention of the activation and clonal expansion of CD4^+^ Teff cells in the MLNs—which, in consequence, prevents the infiltration of the lungs with these cells—constitutes a fundamental event underlying the anti-asthmatic effect induced by all the tested therapeutic strategies. In light of this, it can be stated that all the tested therapeutic strategies counteracted IL-4 and IL-17 production in an indirect fashion, i.e., through the inhibition of the clonal expansion of their primary producers, such as Th2 and Th17 cells, in the MLNs. Additionally, the blockade of NF-κB translocation and of RANKL/RANK interaction prevented the OVA-induced increase in the percentage of IL-4- and IL-17-producing CD4^+^ Teff cells in the lungs. Such results indicate that these therapeutic strategies directly decreased IL-4 and IL-17 production by Th2 and Th17 cells, respectively. This finding strongly suggests that both these therapeutic strategies have the potential to break the pathogenesis of allergic asthma at its later stage by the direct inhibitory effect on the production of two cytokines playing a crucial role [10,11] in promoting the development of this disease. In light of the above, one can assume that if the blockade of NF-κB translocation and of RANKL/RANK interaction does not effectively stop the development of allergic asthma at the level of the clonal expansion of CD4^+^ Teff cells in the MLNs (i.e., in an inductive site of pulmonary immune response), then it can attenuate it at the pulmonary level (i.e., in an effector site of pulmonary immune response) via the inhibitory effect on IL-4- and IL-17 production by lung infiltrating Th2 and Th17 cells, respectively.

In the present study, immunization caused lung infiltration with Tc2 and Tc17 cells. All the tested therapeutic strategies fully prevented this effect. These results suggest that one of the elements contributing to the anti-asthmatic efficacy of these strategies could be the inhibition of Tc2 and Tc17 cell recruitment into the lower respiratory tract. However, none of the tested therapeutic strategies affected the frequency of CD8^+^ T cells capable of IL-4 and IL-17 production. Such results indicate that these strategies do not exert a direct effect on the capacity of CD8^+^ T cells to produce IL-4 and IL-17.

We also verified the hypothesis that the tested therapeutic strategies could induce the production of IL-10 and TGF-β (i.e., crucial anti-inflammatory and immunosuppressive cytokines) by Treg cells and thereby counteract the development of allergic asthma. However, the blockade of IKK, NF-κB translocation or of RANKL/RANK interaction as well as the glucocorticosteroid treatment did not increase both the absolute number and frequency of IL-10- and TGF-β-producing Treg cells, and hence they lacked the potential to counteract the development of the disease via this mechanism.

Additionally, we determined the effect of all the tested therapeutic strategies on IL-10-producing non-Treg CD4^+^ T cells. Immunization not only expanded these cells in the MLNs, but also induced their capacity to produce IL-10 in the lungs. Similarly to Th2 and Th17 cells, all the tested therapeutic strategies indirectly counteracted lung infiltration by IL-10-producing non-Treg CD4^+^ T cells. Moreover, the blockade of NF-κB translocation and of RANKL/RANK interaction prevented the OVA-induced increase in the frequency of IL-10-producting cells among the non-Treg CD4^+^ T cell subset. In OVA-immunized mice, the primary producers of IL-10 within the non-Treg CD4^+^ T cell subset are Th2 cells and inducible type 1 regulatory cells [24]. These subsets were not distinguished in the study, but it seems to be of little importance in the context of this study. The results indicate that none of all the tested therapeutic strategies showed the potential to counteract the development of allergic asthma by increasing the production of IL-10 by CD4^+^ T cells other than Treg ones.

Interestingly, the inhibition of NF-κB activation via the blockade of IKK, same as previously through the administration of glucocorticosteroids (which also act as repressors of NF-κB activity), did not prevent the OVA-induced increase in the percentage of IL-4-, IL-10 and IL-17-producing non-Treg CD4+ T cells (except glucocorticosteroids for IL-17-producing cells). Thus, in the context of asthma therapy, the blockade of NF-κB translocation and of RANKL/RANK interaction seems to have a certain advantage over: (a) the blockade of IKK with regard to the inhibitory effect on IL-4 and IL-17 production in Th2 and Th17 cells, respectively; (b) glucocorticosteroids with regard to the inhibitory effect on IL-4 production in Th2 cells. Furthermore, these results implicate that in the case of some actions, the mechanism of inhibition of NF-κB activity matters whether or not a given effect will appear.

## 4. Materials and Methods

### 4.1. Animals

All the procedures were approved by the Local Ethics Commission (The Local Ethics Commission for Animal Experiments in Olsztyn; Ethical permission No. 10/2017). The experiments were carried out on 6-week-old female Balb/c mice. Mice were maintained under standard laboratory conditions (12/12 h light/dark cycle, controlled temperature (21 ± 2 °C) and humidity (55 +/− 5%), and with *ad libitum* access to autoclaved food and water) in the Animal Facility of the Faculty of Veterinary Medicine, University of Warmia and Mazury in Olsztyn. Mice were euthanized by asphyxiation with CO_2_.

### 4.2. Antigen Immunization, Airway Challenge and Treatment Protocol

**Figure 7:**
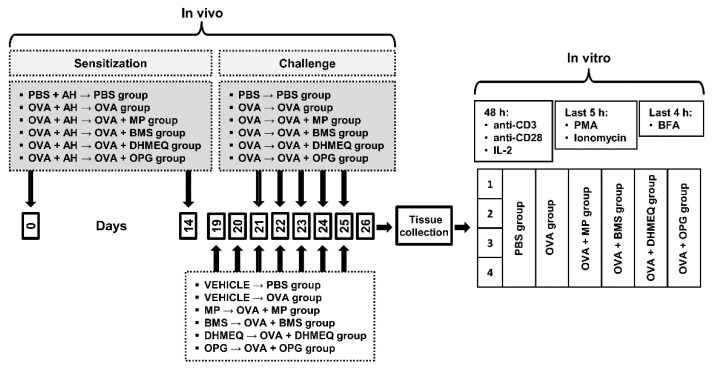
Experimental study design. Mice were divided into six groups, namely the PBS group (PBS-treated and -challenged mice, i.e., healthy mice/negative control), OVA group (ovalbumin (OVA)-sensitized and -challenged mice, i.e., mice with an OVA-induced model of allergic asthma/positive control), OVA + MP, OVA + BMS, OVA + DHMEQ and OVA + OPG groups (i.e., OVA-sensitized and -challenged mice treated with methylprednisolone (MP) or BMS-345541 (BMS) or dehydroxymethylepoxyquinomicin (DHMEQ) or osteoprotegerin (OPG)). Mice were sensitized to OVA by two intraperitoneal (i.p.) injections on days 0 and 14 with OVA absorbed on aluminum hydroxide (AH). Subsequently, mice were challenged intranasally with OVA (OVA, OVA + MP, OVA + BMS, OVA + DHMEQ and OVA + OPG groups) once daily on days 21–28. Mice in the negative control group (PBS group) received only AH in PBS (sensitization) or PBS alone (challenge). MP, BMS, DHMEQ, OPG and vehicles administration was started 48 h prior to the first challenge (i.e., on day 19 after the initial sensitization) and continued daily for 6 consecutive days; all studied agents and/or vehicles were given 3 h before OVA challenge. Mice were euthanized 24 h after the last administration of MP, BMS, DHMEQ, OPG or vehicles, and the mediastinal lymph nodes (MLNs) and lungs were harvested. Lymphocytes isolated from the MLNs and lungs were in vitro activated with plate-coated anti-CD3 and soluble anti-CD28 monoclonal antibodies in the presence of IL-2 for 48 h and re-stimulated with phorbol-12-myristate-13-acetate (PMA) and ionomycin for the last 5 h. Brefeldin A (BFA) was added for final 4 h of culture to inhibit cytokine release by cells. Subsequently, cells were harvested, stained extracellular, fixed, permeabilized, and then stained for intracellular production of IL-4, IL-10, IL-17 and TGF-β.

A schematic diagram showing the design of the experiment is presented in Figure 7. The mice were sensitized and challenged with OVA as previously described [1] and now outlined in Figure 7. The experimental design included the following groups: (I) control/reference groups: (1) the PBS group (PBS-treated and -challenged mice, i.e., healthy mice/negative control), (2) OVA group (OVA-sensitized and -challenged mice, i.e., mice with OVA-induced AAI, which is identified as a model of allergic asthma), and (3) OVA + methylprednisolone (MP) group (OVA-sensitized and -challenged mice treated with glucocorticosteroids with proven efficacy in treating allergic asthma); (II) experimental groups: (4) OVA + BMS (OVA-sensitized and -challenged mice treated with an inhibitor of IKK), (5) OVA + DHMEQ (OVA-sensitized and -challenged mice treated with an inhibitor of NF-κB translocation), and (6) OVA + OPG (OVA-sensitized and -challenged mice treated with an antagonist of RANKL).

MP (Solu-Medrol, Pfizer Manufacturing Belgium NV, Puurs, Belgium) and OPG (Recombinant Mouse Osteoprotegerin/TNFRSF11B Fc Chimera, CF: R&D Systems, Minneapolis, MN, USA) were dissolved in PBS and administered intraperitoneally (i.p.) in doses of 5 mg/kg/day and 0.5 mg/kg/day, respectively. BMS (BMS-345541; Tocris Bioscience, Bristol, UK) and DHMEQ (the reagent was synthesized by TriMenChemicals S.A, Łódź, Poland) were solubilized in dimethyl sulfoxide (DMSO; Sigma-Aldrich, Schnelldorf, Germany) and administered i.p. in doses of 50 mg/kg/day and 25 mg/kg/day, respectively. Mice from OVA + MP and OVA + OPG groups were additionally treated with an equivalent volume of DMSO used as a vehicle to dissolve BMS and DHMEQ; mice from OVA + BMS and OVA + DHMEQ groups were additionally treated with an equivalent volume of PBS used as a vehicle to dissolve MP and OPG; mice from PBS and OVA groups were treated with equal volume mixture of both vehicles. MP, BMS, DHMEQ, OPG and vehicles administration was started 48 h prior to the first challenge (i.e., on day 19 after the initial sensitization) and continued daily for 6 consecutive days; all studied agents and/or vehicles were given 3 h before OVA challenge. MP [25], BMS [26,27], DHMEQ [28,29] and OPG [30,31] doses were chosen according to relevant published reports and our preliminary studies.

### 4.3. Isolation of Inductive and Effector Site Lymphocytes and Culture Conditions

#### 4.3.1. MLNs

MLNs were removed and subjected to Dounce homogenization. The resulting cell suspensions were filtered through Nitex fabric (Fairview Fabrics, Hercules, CA, USA), washed in complete medium (CM; RPMI-1640 +10% heat-inactivated fetal bovine serum +10 mM HEPES (4-(2-hydroxyethyl)-1-piperazineethanesulfonic acid) buffer +10 mM non-essential amino acids +10 mM sodium pyruvate +10 U/mL penicillin/streptomycin; all from Sigma-Aldrich), and centrifuged (300× *g* for 5 min at 5 °C; the same parameters were used for all cell-washing procedures). Cells were re-suspended in CM, counted and adjusted to a final concentration of 4 × 10^6^ cells/mL in CM, and seeded in 24-well plates in 1 mL aliquots.

#### 4.3.2. Lungs

The whole lavaged lungs were minced, subjected to Dounce homogenization, and washed in incomplete medium (RPMI-1640 +10 mM HEPES buffer +10 U/mL penicillin/streptomycin; all from Sigma-Aldrich). Lung lymphocytes were isolated by enzymatic digest (collagenase type IV, 50 U/mL; Sigma-Aldrich) and enriched by density gradient centrifugation (Percoll, Sigma-Aldrich), as previously described [6]. The resulting cell suspensions were filtered through Nitex fabric and washed in CM. Cells were re-suspended in CM, counted and adjusted to a final concentration of 1 × 10^6^ cells/mL in CM, and seeded in 24-well plates in 1 mL aliquots.

#### 4.3.3. Ex Vivo Stimulation of Cytokine Production

As in similar studies [32,33], to detect intracellular cytokines, cells were ex vivo stimulated. Briefly, MLN and lung cells, isolated as described above, were activated with plate-coated anti-CD3 (Purified NA/LE hamster anti-mouse CD3e, 1 μg/mL, clone 145-2C11) and soluble anti-CD28 (Purified NA/LE hamster anti-mouse CD28, 1 μg/mL, clone 37.51) in the presence of IL-2 (Recombinant mouse IL-2, 20 ng/mL; all reagents from BD Biosciences, San Jose, CA, USA) for 48 h. The cells were re-stimulated with phorbol-12-myristate-13-acetate (50 ng/mL) and ionomycin (1 µg/mL; both from Sigma-Aldrich) for the last 5 h. Brefeldin A (Protein transport inhibitor, 1 µL/mL; BD Biosciences) was added for final 4 h of culture to inhibit cytokine release by cells. Cells collected from the MLNs and lungs of vehicle-treated, non-immunized mice were used as the non-stimulated cell culture control. The plates were incubated at 37 °C in an atmosphere of a humidified incubator with 5% CO_2_ and 95% air.

### 4.4. Flow Cytometry

#### 4.4.1. Extracellular Staining

Cells were removed from the wells by pipetting and rinsing with FACS buffer (FB, 1 × Dulbecco’s phosphate-buffered saline (PBS) devoid of Ca^2+^ and Mg^2+^ with 2% (*v*/*v*) heat-inactivated FBS; both reagents from Sigma-Aldrich), transferred into individual tubes and centrifuged. After washing in 2 mL FB, the cells were resuspended in 200 μL FB and pre-treated with anti-CD16/CD32 (clone 2.4G2; BD Biosciences) FcR blocker for 15 min on ice. Subsequently, the cells were stained for surface antigens with fluorochrome-conjugated monoclonal antibodies (mAbs): PerCP-Cy 5.5 rat anti-mouse CD4 (clone RM4-5, IgG2a, κ), APC-Cy7 rat anti-mouse CD8a (clone 53-6.7, IgG2a, κ) and PE-Cy7 rat anti-mouse CD25 (clone PC61, IgG1, λ; all from BD Biosciences). After 45 min incubation (on ice and in the dark), the cells were washed in 2 mL of FB.

#### 4.4.2. Intracellular Staining

Following surface staining, the cells were washed, fixed and permeabilized using Cytofix/Cytoperm solution and Perm/Wash buffer (both from BD Biosciences) according to the manufacturer’s protocol. Subsequently, the cells were stained for Foxp3 expression and intracellular cytokine production with the following fluorochrome-conjugated mAbs: Alexa Fluor 488 rat anti-mouse Foxp3 (clone MF23, IgG2b), PE-CF594 rat anti-mouse IL-4 (clone 11B11, IgG1), APC rat anti-mouse IL-10 (clone JES5-16E3, IgG2b), Alexa Fluor 700 rat anti-mouse IL-17 (clone TC11-18H10, IgG1, λ) and PE mouse anti-mouse TGF-β (LAP; clone TW7-16B4, IgG1, λ; all from BD Biosciences). After 45 min incubation (at room temperature and in the dark), the cells were washed twice with 2 mL of FB and analyzed by flow cytometry.

### 4.5. FACS Acquisition and Data Analysis

**Figure 8:**
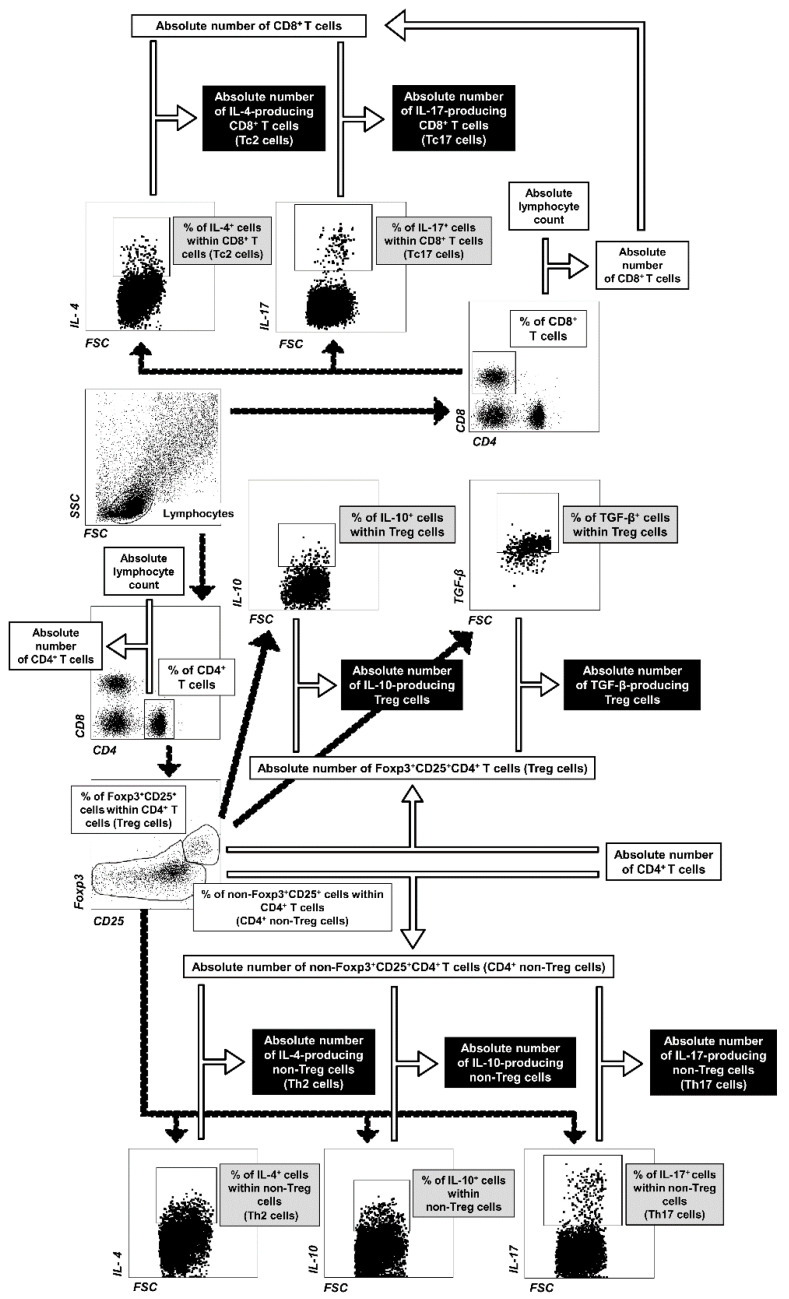
Gating strategy for flow cytometric data analysis and calculation of the absolute cell counts of lymphocyte subsets. Lymphocytes were identified based on forward and side scatter (FSC/SSC) properties, and then gated for expression of CD4 or CD8 surface receptors. CD4^+^ T cells were analyzed for expression/co-expression of CD25 and Foxp3. On this basis, Treg (Foxp3^+^CD25^+^CD4^+^) and non-Treg (the remaining CD4^+^ T cells, i.e., non-Foxp3^+^CD25^+^ CD4^+^ T cells) cells were distinguished. Subsequently, IL-4-, IL-10-, IL-17- and TGF-β-producing cells were identified within particular cell subsets. Absolute cell counts of lymphocyte subsets (i.e., number of cells from particular subpopulations per mediastinal lymph node or lung sample) were calculated using the dual platform method, as shown above.

Flow cytometry analysis was performed using a FACSCelesta cytometer (BD Biosciences). The data were acquired by FACSDiva version 6.1.3 software (BD Biosciences) and analyzed by FlowJo software (Tree Star Inc., Stanford, CA, USA). Absolute cell count was obtained using the dual platform method. Briefly, the total cell count was calculated (using a cell counting chamber) for the MLNs and lungs harvested from individual mice. The absolute cell counts of lymphocyte subsets were determined by recalculating these data by the percentage of particular cell subsets (data from flow cytometry analysis), as illustrated in Figure 8. Thus, the absolute count represented the number of cells from a particular subset per MLNs or lungs collected from individual mice. Fluorescence minus one (FMO) staining and non-stimulated cell control were used to confirm the gating strategy used to identify cytokine-producing cells.

### 4.6. Statistical Analyses

Results were expressed as the mean (±S.D.) of two independent experiments with 4 mice per group (*n* = 8 per group). Statistical analysis was performed using one-way analysis of variance followed by the Bonferroni’s post hoc test. Differences were deemed significant when the *p* values were <0.05. SigmaPlot Software Version 12.0 (Systat Software Inc., San Jose, CA, USA) was used for statistical analysis and the plotting of graphs.

## 5. Conclusions

All the tested therapeutic strategies prevented the OVA-induced increase in the absolute number of Th2 and Th17 cells indirectly, i.e., through the inhibition of the clonal expansion of these cells in the MLNs. Additionally, the blockade of NF-κB translocation and of RANKL/RANK interaction prevented the OVA-induced increase in the frequency of CD4+ T cells capable of IL-4, IL-10 and IL-17 production. These latter results strongly suggest that both therapeutic strategies can directly decrease IL-4 and IL-17 production by Th2 and Th17 cells, respectively. This action may constitute an additional anti-asthmatic mechanism acting as ‘a safety fuse’ inhibiting the development of the disease in case it gets out of control at the level of the inductive site of pulmonary immune response. The inhibition of NF-κB by IKK inhibitor did not affect the percentage of IL-4- and IL-17-producing CD4+ T cells. Thus, in the present context, the blockade of NF-κB translocation and of RANKL/RANK interaction seems to have an advantage over this strategy. Furthermore, these results point out that the type of pharmacological manipulation through which the NF-κB activity is suppressed is important for the occurrence of certain effects associated with the inhibition of this factor. None of the tested therapeutic strategies increased both the absolute number and frequency of IL-10- and TGF-β-producing Treg cells, and hence they lacked the potential to inhibit the development of the disease via this mechanism.

## Data Availability

The data presented in this study are available on reasonable request from the corresponding author.

## Sample Availability

Samples of the compounds are not available from the authors.
